# The effect of linkage disequilibrium on the evolution of resistance to multiple pesticides

**DOI:** 10.1093/jee/toag228

**Published:** 2026-07-27

**Authors:** Luna Q Li, Ricardo Kanitz, Philip Madgwick

**Affiliations:** Department of Biochemistry, University of Oxford, Oxford OX1 3QU, UK; Syngenta Crop Protection, Basel CH-4058, Switzerland; Syngenta, Jealott’s Hill International Research Centre, Bracknell RG42 6EY, UK

**Keywords:** insecticide, resistance, mixtures, simulations, analytics

## Abstract

Pesticides play a crucial role in agricultural pest management and crop yield preservation, yet their extensive application has led to rapid resistance evolution among pest populations. To address this challenge, insecticide resistance management (IRM) strategies aim to prevent or delay resistance evolution. Among the proposed IRM strategies, mixtures are promising but controversial. A key concern with mixtures is the potential for the accelerated evolution of multiple resistance, where selection builds linkage disequilibrium (LD) that leads to a disproportionate number of individuals carrying resistant alleles to both pesticides. In this study, extensive simulations are used to assess the impact of LD on mixtures as an IRM strategy across stochastic and parameter variation. The results demonstrate that LD does not accelerate resistance evolution for mixtures, and analytical predictions explain why resistance alleles are expected to spread independently, even when two pesticides are concurrently applied. These findings suggest that pesticide mixtures can serve as an effective resistance management strategy, outperforming the alternative rotations strategy, particularly when pesticide efficacies are high and exposure is incomplete.

## Introduction

Pesticides are a key tool for pest control in agriculture and public health, but their effectiveness is undermined by the rapid evolution of resistance (Gould et al. 2018). Resistance management aims to develop strategies to delay such evolution to preserve the efficacy of existing pesticides. Early work has unveiled that using multiple pesticides is an effective method for delaying the spread of resistance (Curtis 1985, Mani 1985, Comins 1986, Roush 1989). Mixtures were initially proposed as the superior insecticide resistance management (IRM) strategy (Mani 1985), but further analyses suggested that it is only preferable under a set of restricted conditions (Comins 1986, Roush and McKenzie 1987, Roush 1989). A consensus arose for the set conditions where mixtures outperform other IRM strategies (Tabashnik 1989, Denholm and Rowland 1992, Curtis et al. 1993, Roush 1993, Cloyd 2010, Madgwick and Kanitz 2022b): (1) resistance is initially rare (Comins 1986, Curtis and Otoo 1986, Roush 1998), (2) cross-resistance does not occur (Mani 1985, Caprio 1998), (3) resistance is functionally recessive (Curtis 1985, Curtis et al. 1993), (4) mortality on homozygote susceptible individuals is high (Roush and McKenzie 1987, Roush 1989), and (5) exposure is incomplete (Mani 1985, Curtis and Otoo 1986). Fundamentally, these conditions help mixtures to delay resistance evolution because they facilitate “redundant kill”—where individuals resistant to only one pesticide can be eliminated by the other (Curtis 1985, Comins 1986; see also Madgwick and Kanitz 2022a).

In recent years, analyses have found that mixtures can be preferable to other strategies under more relaxed conditions (Levick et al. 2017, Slater et al. 2017, Sudo et al. 2018, Helps et al. 2020, Madgwick and Kanitz 2022b). One explanation for the discrepancy is that recent research more explicitly address whether mixtures are relatively better or worse than rotations (or solo use), rather than whether mixtures lead to an absolutely longer time to resistance (Madgwick and Kanitz 2022a). These studies also tend to explore a wider range of parameter combinations (Levick et al. 2017, Madgwick and Kanitz 2022b). However, the newer models do not always address all of the arguments that have been put forward against the use of mixtures; for instance, none of the newer studies comprehensively addressed the identified problem of linkage disequilibrium (LD) disfavoring the use of mixtures (Madgwick and Kanitz 2021).

Linkage disequilibrium describes the phenomenon where the nonrandom assortment of alleles leads to their frequency distributions deviating from Hardy–Weinberg equilibrium (HWE) in a population (Robbins 1918). Many mechanisms can lead to the emergence of LD. “Genetic linkage” (Castle 1919, Sturtevant et al. 1919), specifically that alleles residing on the same chromosome interfere with each other’s selection (Hill and Robertson 1966), is one of the many possible causes of LD. It is also possible to generate LD through epistasis where the fitness advantage conferred by one allele depends on the existence of other alleles in the same individual (Wright 1931). Interestingly, applying mixtures as an IRM strategy generates epistasis between the two resistance alleles not because of intrinsic genetic association, but rather as a consequence of environmental correlation (Madgwick and Kanitz 2022b). For an individual resistant to pesticide A, whether it is beneficial to be resistant to pesticide B depends on its location: if it falls in the area where mixtures have been applied, being resistant to pesticide B is beneficial; if it falls in the refuge, being resistant to pesticide B is not beneficial, if not detrimental.

Linkage disequilibrium can act in either the positive or negative direction. In the case of resistance management, positive LD indicates that the double-resistant genotype occurs more frequently than expected from HWE given individual resistance allele frequencies, whereas negative LD indicates a lower double-resistance frequency than expected from HWE. Positive LD was first raised as a potential issue for the use of mixtures for resistance management by Curtis (1985). Curtis argued that if both resistance alleles were effectively dominant, then the rate of selection for the double-heterozygote-resistant genotype (with fully restored fitness) would be faster than the rate at which recombination can break down double-resistance, establishing positive LD. Although no further analysis was provided on the extent of LD this process may generate, the existence of LD would lead to more rapid spread of double-resistance, which was suggested to annihilate the benefits of mixtures. Mani’s work in the same year tackled this issue from a different perspective, where he argued that with sufficiently high recombination, any initial LD can be rapidly resolved (Mani 1985). However, this work explicitly does not address the possibility of positive LD being built up by selection, which was recognized as decreasing the benefits of mixtures. Comins provided a more intricate analysis of the conditions facilitating double-resistance spread in his 1986 paper (Comins 1986). Using analytical derivations, Comins showed that if the multiplicative fitness of the single-resistance haplotypes is lower than half of the fitness of the double-resistance haplotype (assuming free recombination), then selection builds up positive LD, which limits the benefits of mixtures. Therefore, while all of these authors agree that LD can influence the trajectory of resistance evolution under certain conditions when mixtures are applied, there remains little consensus on the precise criteria necessary or the degree to which positive LD accelerates resistance selection.

A major difficulty in assessing the importance of LD on resistance evolution is that all three models are deterministic. The concern that positive LD imposes on mixtures as an effective IRM strategy hinges on the evolving population’s ability to produce individuals resistant to both pesticides. With both resistance alleles beginning at low frequencies in the population, the generation of the even rarer double-resistant genotype is dependent on stochasticity bringing the alleles into the same genotype (Felsenstein 1974). Additionally, there is also the possibility of a countervailing effect, where the selection of both single-resistant genotypes in separate backgrounds leads to selective interference and negative LD (Hill and Robertson 1966, Felsenstein 1974). In either the case of positive or negative LD, stochasticity is not a well-highlighted factor in prior analyses. Even in recent years, existing models of resistance evolution model exploring LD employ deterministic dynamics (Levick et al. 2017, Madgwick and Kanitz 2022b), and many more ignore the possibility of LD altogether (Sudo et al. 2018, Helps et al. 2020, Hobbs et al. 2023).

To address these limitations, a computational framework is constructed to provide an explicit and comprehensive answer to the possible effects of LD on the choice of resistance management strategy under stochastic simulations. As a benchmark, mixture results are compared against the performance of per-generation rotations (pesticides A and B used alternatively at each generation). Three related population genetics models are constructed to compare the different effects arising from stochasticity, which influence LD. Extensive simulations of a wide range of parameter combinations are used to systematically assess the differences between the three models. Lastly, analytical equations are developed to accurately predict key simulation outputs to explain the results.

## Methods

The population genetics modeling framework used is based on Wright–Fisher dynamics (Fisher 1930, Wright 1931), assuming discrete and nonoverlapping generations of constant population size. To generate comparable results to prior works, the modeling framework chosen in this study follows the assumptions made by multiple prior computational studies on resistance evolution under pesticide application (Birget and Koella 2015, Levick et al. 2017, South and Hastings 2018, Madgwick and Kanitz 2022b, Hobbs et al. 2023). Within each generation, pesticide selection occurs prior to adult dispersal and random mating, the ordering of which captures a wide range of pests whose juvenile stages are dependent on the crop and therefore exposed to pesticides (Helps et al. 2017, Sudo et al. 2018). Resistance is assumed to be monogenic, with each pesticide having a single locus of major effect with a resistant and susceptible allele. Resistance alleles are assumed not to be biologically linked and freely recombine (*r* = 0.5).

The overall model workflow can be summarized as the following:

- Parameter sets are chosen randomly from a predefined parameter space.
- Initial allele frequencies are adjusted to ensure the existence of at least one individual carrying the resistance allele in the population.
- Parameter sets are filtered for cases where, under solo-pesticide application, positive selection of both resistance alleles occurs.
- The filtered parameter sets are then used as input parameters to the main simulation under three different sets of assumptions (deterministic allelic recombination [DAR], stochastic allelic recombination [SAR], and stochastic genotypic recombination [SGR]).
- The simulation output collected is informative of one of three possible outcomes for the resistance allele (fixation, elimination, spreading) under selection and the generation at which this occurs.

All simulations and calculations are carried out in R version 4.4.1.

### Simulation Input Parameters

Similar to previous models (Levick et al. 2017, Madgwick and Kanitz 2022b), fourteen parameters are included as model inputs, with corresponding ranges listed in Table 1. Specifically, the fourteen parameters include: k—population size, which is constant throughout resistance evolution; x—overall pesticide exposure, which determines the fraction of the population which encounters pesticides at any generation; $f_{A}^{0},\;f_{B}^{0}$—the initial resistance allele frequencies for pesticide A and B, respectively; $m_{A},\;m_{B}$—the effectiveness of pesticide A and B, respectively, indicating the proportion of fully susceptible individuals which be killed by encountering each pesticide; $r_{A},\;r_{B}$—the recovery conferred by resistance allele A and B, respectively; $h_{A},\;h_{B}$—the dominance of resistance allele A and B, respectively; $c_{A},\;c_{B}$—the cost of carrying resistance allele A and B, respectively; $hc_{A},\;hc_{B}$—the dominance of the cost of resistance allele A and B, respectively.

**Table 1.** Simulation parameters and respective ranges

Parameter	Symbol	Range
**Population size**	k	10^2^–10^9^
**Compound exposure**	x	0–1
**Initial resistance allele frequency**	$f_{A}^{0},\;f_{B}^{0}$	10^−9^–10^−2^ (adjusted to $\geq \frac{1}{k}$)
**Compound efficacy**	$m_{A},\;m_{B}$	0–1
**Recovery conferred by resistance allele**	$r_{A},\;r_{B}$	0–1
**Dominance of resistance allele**	$h_{A},\;h_{B}$	0–1
**Cost of resistance allele**	$c_{A},\;c_{B}$	10^-3^–10^−0.5^
**Dominance of cost of resistance allele**	$hc_{A},\;hc_{B}$	0–1

To select an unbiased range of parameter combinations for downstream analysis and cover a wide range of possible scenarios, parameters with values between 0 and 1 are sampled randomly from uniform distributions. For the remaining parameters, the exponents are sampled randomly from uniform distributions. 65,000 of such parameter sets are generated, and an adjustment is performed to ensure that the starting allele frequency for resistance alleles A and B are at least equal to $\frac{1}{k}$. Such adjustment guaranteed that at least one individual carries each resistance allele, regardless of the model assumptions later employed.

Since only resistance alleles with the potential to spread pose a challenge to resistance management, it is necessary to filter the 65,000 parameter sets to select those containing sufficiently beneficial resistance alleles. The 65,000 parameter sets are used as inputs to a preliminary solo-pesticide selection simulation with DAR dynamics (see Methods section on “Deterministic allele recombination…”). One generation of the deterministic simulation is run, and the ending frequency of the allele under selection is retrieved. Only parameter sets where both resistance alleles increase in frequency under respective solo-pesticide selection are accepted, and the first 30,000 of these are chosen as input for the main simulation. The choice of parameter space here prioritizes an unbiased selection of all possible parameter combinations rather than what may constitute an effective resistance management strategy in reality. Consequently, results which summarize observations across the entire parameter space should be interpreted with caution, and readers are referred to the decision tree figures to identify biologically relevant resistance management strategies.

### Mathematical Representation of Selection Mechanisms

Pesticide selection is based on fitness scores calculated from initial input parameters, similar to previous models (Levick et al. 2017, Madgwick and Kanitz 2022b). For each input parameter set, three fitness scores are calculated for each of the nine possible genotypes. The fitness scores describe the expected survival of each genotype under solo pesticide A exposure, solo pesticide B exposure, and combined A & B exposure. The first two of the fitness scores are used in alternate generations to simulate selection pressures imposed by rotating pesticides A and B on a per-generation basis, and the third is representative of mixture selection under a multiplicative assumption (Madgwick and Kanitz 2021). The exact formulae are shown in Table 2, with resistant allele genotypes being represented with uppercase letters A and B, and the susceptible equivalents in lower case. It should be noted that the resistance management strategies adopted represent idealized implementations of mixtures and rotations, as the model does not fully capture the complexity of seasonal dynamics and spray timing in real-world conditions.

**Table 2.** Mathematical representations of genotype fitness, for similar fitness representations see Table 2 and Table S1 of Levick et al. (2017) and Table 2 of Madgwick and Kanitz (2022b) (Levick et al. 2017, Madgwick and Kanitz 2022b)

	Pesticide A selection	Pesticide B selection	Mixture selection
$\omega_{\mathrm{AABB}}$	$\left((1-x)+x(1-m_{A}(1-r_{A})))\times (1-c_{A})\times (1-c_{B}\right)$	$\left((1-x)+x(1-m_{B}(1-r_{B})))\times (1-c_{A})\times (1-c_{B}\right)$	$\left((1-x)+x(1-m_{A}(1-r_{A})))\times (1-m_{B}(1-r_{B}))\times (1-c_{A})\times (1-c_{B}\right)$
$\omega_{\mathrm{AaBB}}$	$((1-x)+x(1-m_{A}(1-h_{A}r_{A})))\times (1-hc_{A}c_{A})\times (1-c_{B})\;$	$\left((1-x)+x(1-m_{B}(1-r_{B})))\times (1-hc_{A}c_{A})\times (1-c_{B}\right)$	$\left((1-x)+x(1-m_{A}(1-h_{A}r_{A}))\times (1-m_{B}(1-r_{B})))\times (1-hc_{A}c_{A})\times (1-c_{B}\right)$
$\omega_{\mathrm{aaBB}}$	$\left((1-x)+x(1-m_{A}))\times (1-c_{B}\right)$	$\left((1-x)+x(1-m_{B}(1-r_{B})))\times (1-c_{B}\right)$	$\left((1-x)+x(1-m_{A})\times (1-m_{B}\times (1-r_{B})))\times (1-c_{B}\right)$
$\omega_{\mathrm{AABb}}$	$\left((1-x)+x(1-m_{A}(1-r_{A})))\times (1-c_{A})\times (1-hc_{B}c_{B}\right)$	$\left((1-x)+x(1-m_{B}(1-h_{B}r_{B})))\times (1-c_{A})\times (1-hc_{B}c_{B}\right)$	$\left((1-x)+x(1-m_{A}(1-r_{A}))\times (1-m_{B}(1-h_{B}r_{B})))\times (1-c_{A})\times (1-hc_{B}c_{B}\right)$
$\omega_{\mathrm{AaBb}}$	$\left((1-x)+x(1-m_{A}(1-h_{A}r_{A})))\times (1-hc_{A}c_{A})\times (1-hc_{B}c_{B}\right)$	$\left((1-x)+x(1-m_{B}(1-h_{B}r_{B})))\times (1-hc_{A}c_{A})\times (1-hc_{B}c_{B}\right)$	$\left((1-x)+x(1-mA(1-h_{A}r_{A}))\times (1-mB(1-h_{B}r_{B})))\times (1-hc_{A}c_{A})\times (1-hc_{B}c_{B}\right)$
$\omega_{\mathrm{aaBb}}$	$\left((1-x)+x(1-m_{A}))\times (1-hc_{B}c_{B}\right)$	$\left((1-x)+x(1-m_{B}(1-h_{B}r_{B})))\times (1-hc_{B}c_{B}\right)$	$\left((1-x)+x(1-m_{A})\times (1-m_{B}(1-h_{B}r_{B})))\times (1-hc_{B}c_{B}\right)$
$\omega_{\mathrm{AAbb}}$	$\left((1-x)+x(1-m_{A}(1-r_{A})))\times (1-c_{A}\right)$	$\left((1-x)+x(1-m_{B}))\times (1-c_{A}\right)$	$\left((1-x)+x(1-m_{A}(1-r_{A}))\times (1-m_{B}))\times (1-c_{A}\right)$
$\omega_{\mathrm{Aabb}}$	$\left((1-x)+x(1-m_{A}(1-h_{A}r_{A})))\times (1-hc_{A}c_{A}\right)$	$\left((1-x)+x(1-m_{B}))\times (1-hc_{A}c_{A}\right)$	$\left((1-x)+x(1-m_{A}(1-h_{A}r_{A}))\times (1-m_{B}))\times (1-hc_{A}c_{A}\right)$
$\omega_{\mathrm{aabb}}$	$\left((1-x)+x(1-m_{A})\right)$	$\left((1-x)+x(1-m_{B})\right)$	$\left((1-x)+x(1-m_{A})\times (1-m_{B})\right)$

### DAR, SAR, and SGR Models

Starting from the same foundational dynamics, three parallel models are constructed to understand the effects of linkage disequilibrium (LD) on resistance allele selection under stochastic dynamics. Two components of LD, namely the effects of stochasticity and gene assortment, are assessed separately by using control models that lack one or both of these components. The overall effect of LD is then determined by comparing these control models with a full model which incorporates both components, which more closely reflects biological reality.

#### DAR Model

The DAR model follows existing population genetics models previously constructed to simulate pesticide resistance evolution (Levick et al. 2017, Madgwick and Kanitz 2022b). At the start of the simulation, the distribution of the nine possible genotypes at a population level is calculated from $f_{A}^{0}$ (starting resistance allele A frequency) and $f_{B}^{0}$ (starting resistance allele B frequency) assuming HWE where the frequency distributions of the two resistance alleles in the population are mutually independent and neither positive nor negative linkage exists. The simulation proceeds to select upon this population without stochasticity, and the population-level allele frequencies of resistance alleles A and B are collected at the end of each generation. This collective frequency is then used to calculate the frequency of the nine genotypes at the beginning of the next generation, assuming HWE.

#### SAR Model

The SAR model is an extension of the DAR model. Similar to DAR, the frequency distribution of the nine genotypes is always constrained by HWE, but unlike the DAR model, the SAR model incorporates stochasticity. At the beginning of each generation, the collective population-level resistance allele frequencies are partitioned to each of the nine genotypes assuming HWE. Selection proceeds to act on the population, mating occurs, and stochasticity is introduced following zygote formation to represent reproductive randomness. The distribution of the nine possible genotypes is then sampled using a multinomial distribution. To keep the population at a constant size, the multinomial sampling process assumes that k offspring will be born. The sampled genotypic frequencies are then collected, as in DAR, to give the total population-level frequency of resistance alleles A and B. These collective frequencies are passed on to the next generation. To explore the full range of stochastic outcomes, 10,000 simulation repeats are run for each parameter set; this is then applied to both mixtures and per-generation rotations, resulting in a total of 600,000,000 individual simulations for the SAR model.

#### SGR Model

The SGR model is an extension of the SAR model, and it most closely resembles genetic systems in reality. The implementation of stochasticity remains the same as in SAR; however, genotypic distributions are allowed to deviate from HWE. In the first generation, resistance alleles begin exclusively in heterozygotes with genotype Aabb or aaBb at a frequency of $2\times f_{A}^{0}$ and $2\times f_{B}^{0}$, respectively, to account for heterozygosity. Under this setup, there is initially weak negative linkage disequilibrium (LD) between the two resistance alleles. However, because resistance alleles are rare at the start of the simulation, double-resistant individuals are highly unlikely to occur in the founding population, the deviation of LD from zero is therefore small. Selection and stochastic sampling proceed in the same fashion as in the SAR model, but instead of recording the collective allele frequencies, the exact frequencies of the nine possible genotypes are recorded and used as the starting genotype distribution for the next generation of the simulation. Effectively, resistance genotypes can no longer freely mix at the allelic level, as the parental genotypes recombine through the independent assortment of alleles during meiosis and gamete fusion into the offspring genotypes. Similar to SAR, 10,000 simulation repeats are run for each parameter set and each pesticide regimen under SGR dynamics.

### Simulation Termination and Output

The simulation terminates if both resistance allele frequencies have reached 0 (elimination) or 0.5 (fixation threshold); otherwise, it proceeds until generation 500, at which point the simulation terminates unconditionally. The elimination or fixation of one resistance allele does not alter the strategy employed and does not result in the termination of the simulation. 0.5 is chosen in accordance with common practices in related literature (REX Consortium 2010), and it is unlikely that a different threshold value should impact the conclusions drawn. Five-hundred generations is chosen as the effective endpoint, as it represents a sufficiently long time horizon over which a single pesticide may realistically be deployed (Levick et al. 2017, Madgwick and Kanitz 2022b, Hobbs et al. 2023). A subset of parameters are run using a 0.9 fixation threshold and a 1,000-generation horizon as a sensitivity check.

Two values are collected for each resistance allele when an elimination or fixation event occurs in the simulation: the population-level allele frequency at this point and the total number of generations lapsed. Should a resistance allele not experience an elimination or fixation event by generation 500, its allele frequency at generation 500 is recorded. In total, three categories of simulation outcomes are possible:

#### Fixation

The resistance allele reaches the 0.5 frequency threshold to be considered fixed within the 500-generation timeframe. The simulation records the generation at which resistance fixation occurs and the allele frequency (which must be >0.5) at this generation.

#### Elimination

The resistance allele reaches a frequency of 0 within the 500-generation timeframe to be considered lost. The simulation records the generation at which resistance was lost and the ending resistance allele frequency of 0.

#### Spreading

It is possible for the resistance allele to remain at an intermediate level between 0 and 0.5 at generation 500. In this case, the simulation records the ending generation number, 500, as well as the population-level ending allele frequency, which must be greater than 0 but less than 0.5.

For the SGR model, additional information is collected regarding the status of LD at the final generation prior to the allele fixation threshold being reached. Normalized gamete frequencies ($P_{AB},\;P_{Ab},\;P_{aB},\;P_{ab}$) are computed from the parent generation genotype distribution, taking into account the fitness of each genotype during selection and its current frequency, assuming free recombination during gamete formation, and then normalized to sum to 1. Lewontin’s *D* and normalized *D* (*D*′) are calculated as follows (Lewontin 1964):


$$p_{A}=P_{AB}+P_{Ab},\;p_{B}=P_{AB}+P_{aB},$$

$$D=P_{AB}-p_{A}p_{B},$$

$$D^{'}=\;\frac{D}{D_{\max}},$$

where:

If D>0:


$$D_{\max}=\min\left((1-p_{B}),\;(1-p_{A})p_{B}\right),$$

If D<0:


$$D_{\max}=\min\left(p_{A}p_{B},\;(1-p_{A})(1-p_{B})\right).$$

D′ has a value between −1 and 1. A positive D’ indicates an excess of the AB and ab haplotypes relative to expectations under linkage equilibrium, whereas a negative $D^{\prime }$ indicates an excess of the Ab and aB haplotypes. The nonzero median of each parameter set is reported.

### Analysing Simulation Outputs

Unless otherwise specified, all analyses were performed on model output with regards to resistance allele A.

### Comparing Mixtures and Rotations Within Each Model

Comparisons between mixture and rotation application strategies are made individually for each resistance allele in each model. The goal is to determine which strategy is superior, with resistance allele elimination being the most favorable outcome, followed by spreading and then fixation.

In the DAR model, a direct comparison is made between the deterministic simulation outputs for a given parameter set. If one strategy leads to allele elimination and the other does not, the elimination-producing strategy is deemed superior. If both strategies result in the same outcome category (eg both lead to fixation), the numerical values are compared. For fixation, the strategy with a longer time-to-fixation is superior. For elimination, the strategy with a shorter time-to-elimination is superior. For alleles which are spreading by the end of the simulation, the strategy with a lower final allele frequency is superior. If no strategy can be considered superior after these comparisons, the outcome is registered as “neither.”

In both SAR and SGR models, stochastic effects are accounted for by repeating the comparison multiple times. For the same parameter set, one simulation each is chosen from the 10,000 repeats run for mixtures and rotations randomly, and the same comparison procedure used in the DAR model is applied. This process is repeated 100,000 times to provide a 10-fold coverage of all stochastic repeats. The results of these 100,000 comparisons are then averaged to determine the probability that either mixtures or rotations are the superior strategy for that particular parameter set.

### Summary Tables for Stochastic Simulations

To analyze stochastic simulation outcomes, summary tables are generated to condense the range of possible outcomes into a useful classification. The focus is on cases where resistance alleles are either fixed or eliminated. For each parameter set, four key metrics are calculated: the probability-of-fixation, the probability-of-elimination, the mean time-to-fixation (conditional on fixation), and the mean time-to-elimination (conditional on elimination). These values summarize the overall dynamics of each resistance allele across all 10,000 stochastic simulation repeats for a given parameter combination.

### Decision Tree Analysis

Conditional inference trees are used to identify regions of the parameter space that favor certain simulation outcomes. The ctree function from the “partykit” package (Hothorn and Zeileis 2015) in R creates a tree-like structure by making binary splits in the fourteen input parameters that best differentiate the simulation outcomes. To ensure the tree is interpretable and avoid overfitting, the maximum tree depth is limited to 3, and the minimum bin size is set to 500. One deterministic analysis is carried out on the entire parameter set. Simulation parameters are randomly sampled to ensure an unbiased decision tree analysis. The frequency distribution at each terminal branch is dependent on the choice of parameter space and should therefore be interpreted with caution.

### Analytical Approximation of Key Simulation Outcomes

To analytically approximate key simulation outcome metrics such as time-to-fixation and probability-of-fixation (under stochastic dynamics), the selection coefficient is calculated first and compared with the corresponding simulation-derived value. Selection coefficient calculation from simulation data involves fitting a linear model to transformed starting and ending resistance allele frequency as a function of the number of generations lapsed. Resistance allele frequencies, f, are transformed according to Equation (1). The observed selection coefficient, $s_{\mathrm{obs}}$, is derived from the gradient (g) of the fitted linear model, according to Equation (2):


(1) f'=log10⁡f1-f ,

(2) sobs=10g-1 .

Predicting selection coefficients under a DAR solo-use regimen assumes that the heterozygote resistance fitness ($wAa_{\mathrm{solo}}$) is the main determinant in the rate of resistance allele spread when the allele remains rare (Comins 1977, 1986, May and Dobson 1986). The fitness of the heterozygote resistant individual instead depends on exposure, pesticide efficacy, resistance allele benefit and cost, as well as their respective dominance terms [Equation (3)] (Levick et al. 2017, Madgwick and Kanitz 2022a, Madgwick et al. 2024). Fitness of the susceptible individual ($waa_{\mathrm{solo}}$) is only dependent on exposure and pesticide efficacy [Equation (4)]. The fitness advantage of wAa over waa is related to $s_{\mathrm{solo}}$ through Equation (5):


(3) wAasolo=(1-xmA(1-hArA))×(1-hcAcA) ,

(4) waasolo=1-xmA ,

(5) ssolo=wAasolowaasolo-1 .

In a rotations regimen under DAR assumptions, the fitness of the Aabb genotype ($\mathrm{wAab}b_{\mathrm{rot}}$) is calculated with Equation (6). Fitness of the fully susceptible genotype aabb ($\mathrm{waab}b_{\mathrm{rot}}$) is calculated with Equation (7), and $s_{\mathrm{rot}}$ with Equation (8). The formulae for calculating genotype fitness are related to Equations (3) and (4) in the solo-use case. However, due to pesticide A being applied at every other generation in a rotation regimen, effectively any selection pressure exerted by pesticide A must be spread over two generations. This is accommodated by square-rooting genotype fitness benefit (Madgwick and Kanitz 2022a) and keeping the cost term unchanged as the cost manifests in the genotype’s overall fitness at every generation. A similar set of formulae is used to calculate $s_{\mathrm{mix}}$ in a mixture regimen under DAR model assumptions [Equations (9)–(11)]:


(6) wAabbrot=(1-x)+x(1-mA(1-hArA))×1-xmB×(1-hcAcA) ,

(7) waabbrot=1-xmA×1-xmB ,

(8) srot=wAabbrotwaabbrot-1 ,

(9) wAabbmix=((1-x)+x(1-mA(1-hArA))×(1-mB))×(1-hcAcA) ,

(10) waabbmix=(1-x)+x(1-mA)×(1-mB) ,

(11) smix=wAabbmixwaabbmix-1 .

In the stochastic models, such as SAR and SGR, two key metrics are analytically predicted: probability-of-fixation and time-to-fixation. These predictions utilize the selection coefficient, now generally denoted as s, calculated from DAR analytics above using the appropriate pesticide regimen. Probability-of-fixation ($p_{\mathrm{fix}}$) is calculated using a simplified form of Kimura’s equation (Kimura 1957, Kimura 1962) [Equation (12)]:


(12) pfix=1-e-4sfA0k .

The naïve time-to-fixation prediction is given using Equation (13) which is derived from the previously reported continuous-time allele frequency prediction (Madgwick and Kanitz 2022a). As with probability-of-fixation predictions, the appropriate selection coefficient s as calculated using Equations (3), (6) or (9) is used. An additional adjustment is made for parameter sets where the resistance allele begins at one homozygote copy in the population—the lowest value allowed in the simulation. For these parameter sets, a slight improvement over Equation (13) is possible, which adjusts for the possibility of losing the resistance alleles in the first generation [Equation (14)]. In Equation (14), $p_{\mathrm{fix}}\;$denotes the probability-of-fixation as predicted using Equation (12), and F is the fixation frequency threshold of 0.5:


(13) tfix=ln⁡(1-fA0fA0)ln⁡(1+s) ,

(14) tfix=ln(pfix×F×(1-fA0)(1-pfix×F)×fA0) ln⁡(1+s) .

## Results

Three related population genetics models are used to understand the effects of stochasticity and gene assortment on resistance evolution. 65,000 parameter sets are randomly from a predetermined parameter space, encompassing 14 parameters relevant for pesticide resistance evolution (see Table 1). After filtering for sufficiently beneficial resistance alleles, 30,000 parameter sets are selected and used as input for the three models:

- DAR model: follows deterministic dynamics, with gene assortment at the allelic level assuming HWE.
- SAR model: follows stochastic dynamics, with gene assortment similarly at the allelic level assuming HWE.
- SGR model: follows stochastic dynamics, and gene assortment follows independent assortment as in meiosis and is not constrained by HWE.

Simulations are run for each model using the same starting parameter sets with different application strategies. In the DAR model, one simulation is run per parameter set and per application strategy, totaling 60,000 simulations. For the SAR and SGR models, 10,000 repeats are run per parameter set to explore stochastic outcomes, resulting in 600,000,000 simulations per model.

Each simulation is run for up to 500 generations. A simulation is considered complete when the resistance allele frequency either reached 0.5 (fixed) or 0 (eliminated). If neither of these conditions is met by generation 500, the simulation is terminated and a “spreading” outcome is registered. The terminal generation number and final resistance allele frequency are recorded for each simulation.

### Deterministic Allelic Recombination

Comparing resistance management outcomes for mixtures and rotations under the DAR model, across the entire parameter space, rotations are the superior strategy in 73.86% of all simulations, while mixtures are superior in 25.85% of simulations (Fig. 1A). A small fraction (0.29%) shows no superior strategy (Fig. 1A). The numerical figure should be interpreted with caution, as not all areas of the parameter space can be used as a viable resistance management strategy in field contexts.

**Fig. 1.**
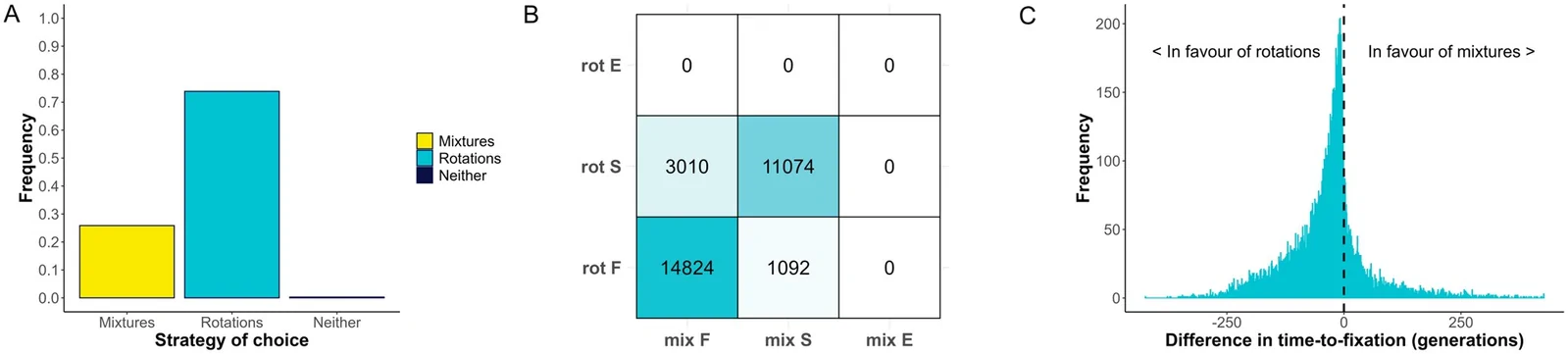
Overview of results from the deterministic allelic assortment (DAR) model. (A) Overall preference for mixtures, rotations, or neither as an IRM strategy across the whole parameter space explored. The numerical value shown is the fraction of parameter sets which fell into each category under the DAR model. (B) The categorical resistance management outcome correlation matrix between mixtures and rotations under the DAR model. F—resistance allele reached fixation threshold of 0.5; S—resistance allele continued to spread at generation 500, it never reached frequencies of 0.5 or 0 within the simulation; E—resistance allele reached a frequency of 0 in the population and was eliminated. The numerical value shown is the absolute number of parameter sets which fell into each category out of all 30,000 parameter sets. (C) Of the 14,824 parameter sets where both mixtures and rotations led to resistance allele fixation under the DAR model, the difference between the number of generations taken for the two strategies to fix the resistance allele.

The model also indicates that resistance alleles are never eliminated from the population (Fig. 1B). There is high correlation between categorical outcomes, with 86.33% of simulations showing the same outcome category for both strategies (Fig. 1B). In cases where both strategies lead to resistance allele fixation (14,824 parameter combinations), rotations are favored for their longer time-to-fixation (Fig. 1C). This is in line with expectations, as mixtures impose a higher selection pressure on resistance alleles, causing resistance alleles to increase in frequency more rapidly. A decision tree analysis reveals that the most important factor underlying strategy choice is $m_{B}$ (the efficacy of pesticide B)—with a higher value favoring mixtures, followed by x (exposure) and $m_{A}$ (efficacy of pesticide A), with low x and high $m_{A}$ favoring mixtures (Fig. 2). A similar pattern is observed when the fixation threshold is 0.9 (Supplementary Fig. S1).

**Fig. 2.**
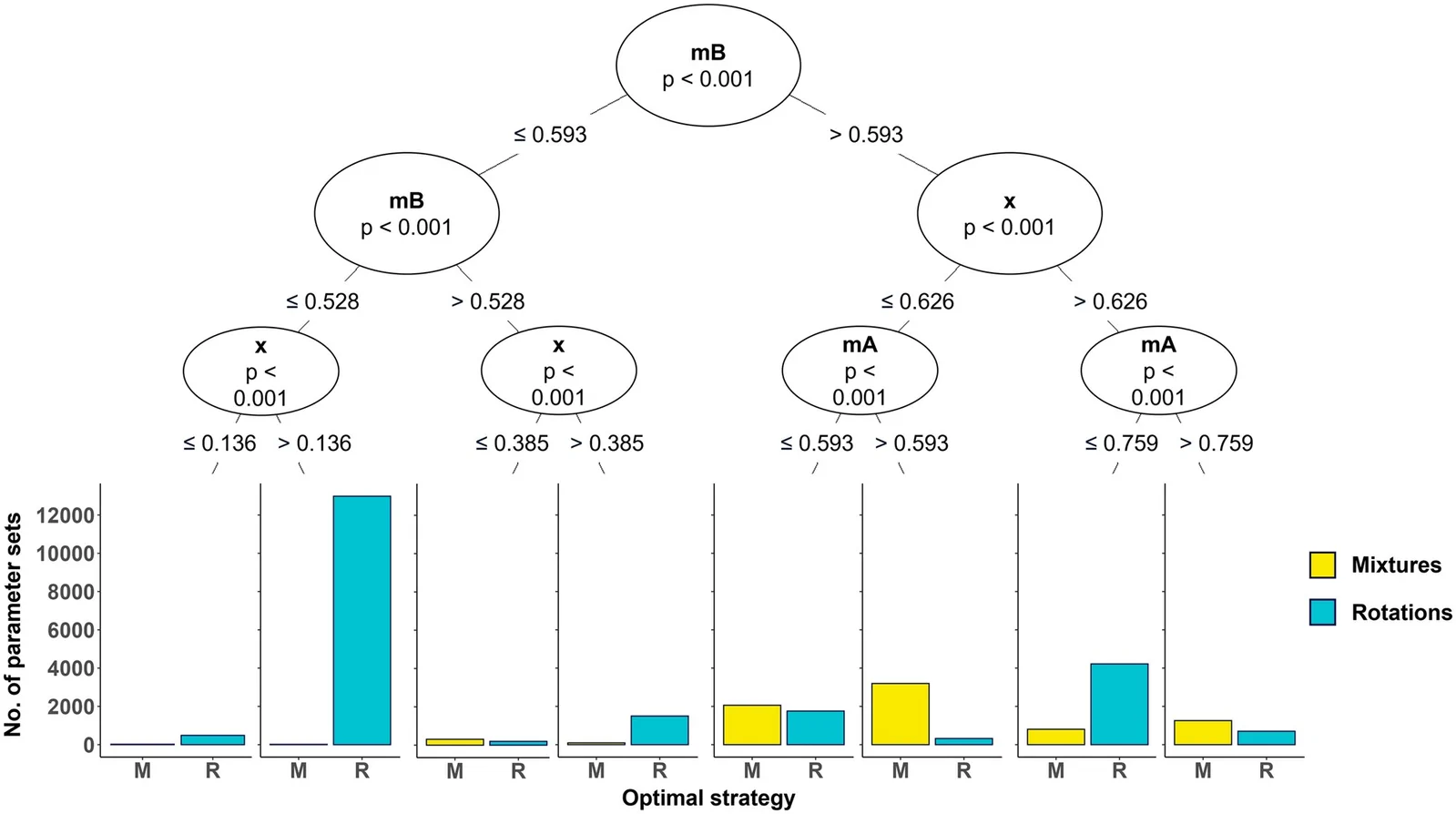
Decision tree analysis of parameter space preference for IRM strategies (mixtures or rotations) under the DAR model. mB—efficacy of pesticide B; x–exposure; mA—efficacy of pesticide A. Values displayed on the bar chart at each leaf node is the absolute number of parameter sets (from a total of 30,000) which fall into the parameter space segment as specified in the tree structure.

### Stochastic Allelic Recombination

The SAR model introduces stochasticity to the DAR model. The SAR model shows rotations to be the superior strategy in 58.43% of all simulations (Fig. 3A), a decrease of 15.43% compared to the DAR model. Mixtures are the superior strategy in 38.39% of simulations (Fig. 3A), a 12.54% increase compared to the DAR model. To take stochastic effects into account, Fig. 3B presents the modal outcome of all stochastic repeats. The most notable difference from the DAR model is that resistance allele can now be eliminated, which is the modal outcome in 51.36% of all simulations (Fig. 3B).

**Fig. 3.**
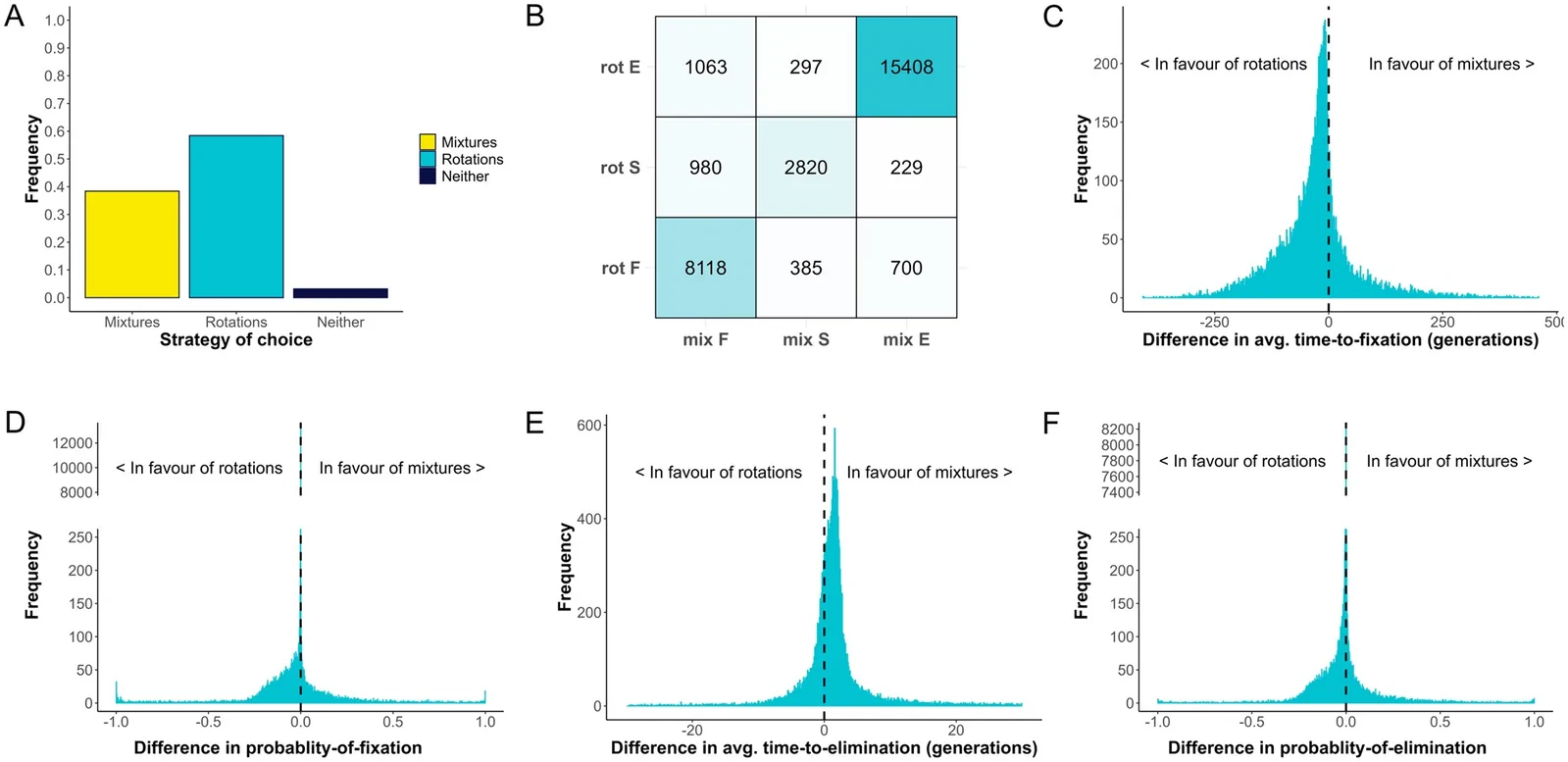
Overview of results from the stochastic allelic assortment (SAR) model. (A) Overall preference for mixtures, rotations, or neither as an IRM strategy across the whole parameter space explored. The numerical value shown is the fraction of parameter sets which falls into each category under the SAR model. (B) The categorical resistance management outcome correlation matrix between mixtures and rotations under the SAR model. F—resistance allele reached fixation threshold of 0.5; S—resistance allele continued to spread at generation 500, it never reached frequencies of 0.5 or 0 within the simulation; E—resistance allele reached a frequency of 0 in the population and was eliminated. The numerical value shown is the absolute number of parameter sets which fell into each category out of all 30,000 parameter sets. (C) Conditional upon fixation, the difference between the number of generations taken for mixtures and rotations to fix the resistance allele under the SAR model. (D) The difference in probability-of-fixation between mixtures and rotations across all 30,000 parameter sets under the SAR model. (E) Conditional upon elimination, the difference between the number of generations taken for mixtures and rotations to eliminate the resistance allele under the SAR model. (F) The difference in probability-of-elimination between mixtures and rotations across all 30,000 parameter sets under the SAR model.

Similar to the DAR model, 87.82% of all parameter combinations result in the same category of resistance management outcome with either strategy (Fig. 3B). Conditional upon fixation, average time-to-fixation favors rotations (Fig. 3C). The addition of stochasticity permits the comparison of probabilistic outcomes. In general, probability-of-fixation and probability-of-elimination both favor rotations (Fig. 3D and F), whereas time-to-elimination strongly favors mixtures (Fig. 3E). A decision tree analysis similar to that in DAR is carried out to understand the most important parameters underlying strategy choice. An additional category is added for cases where neither strategy is able to outperform the other by a margin of least 10%. Similar to DAR, high efficacies and low exposure favor mixtures over rotations (Fig. 4), and remains the case when fixation threshold is increased to 0.9 (Supplementary Fig. S2). However, only two of the eight leaf nodes show a clear preference for either strategy: one favoring rotations (low $m_{B}$, high x, high $m_{A}$), and one favoring mixtures (high $m_{B}$, high $m_{A}$, low x).

**Fig. 4.**
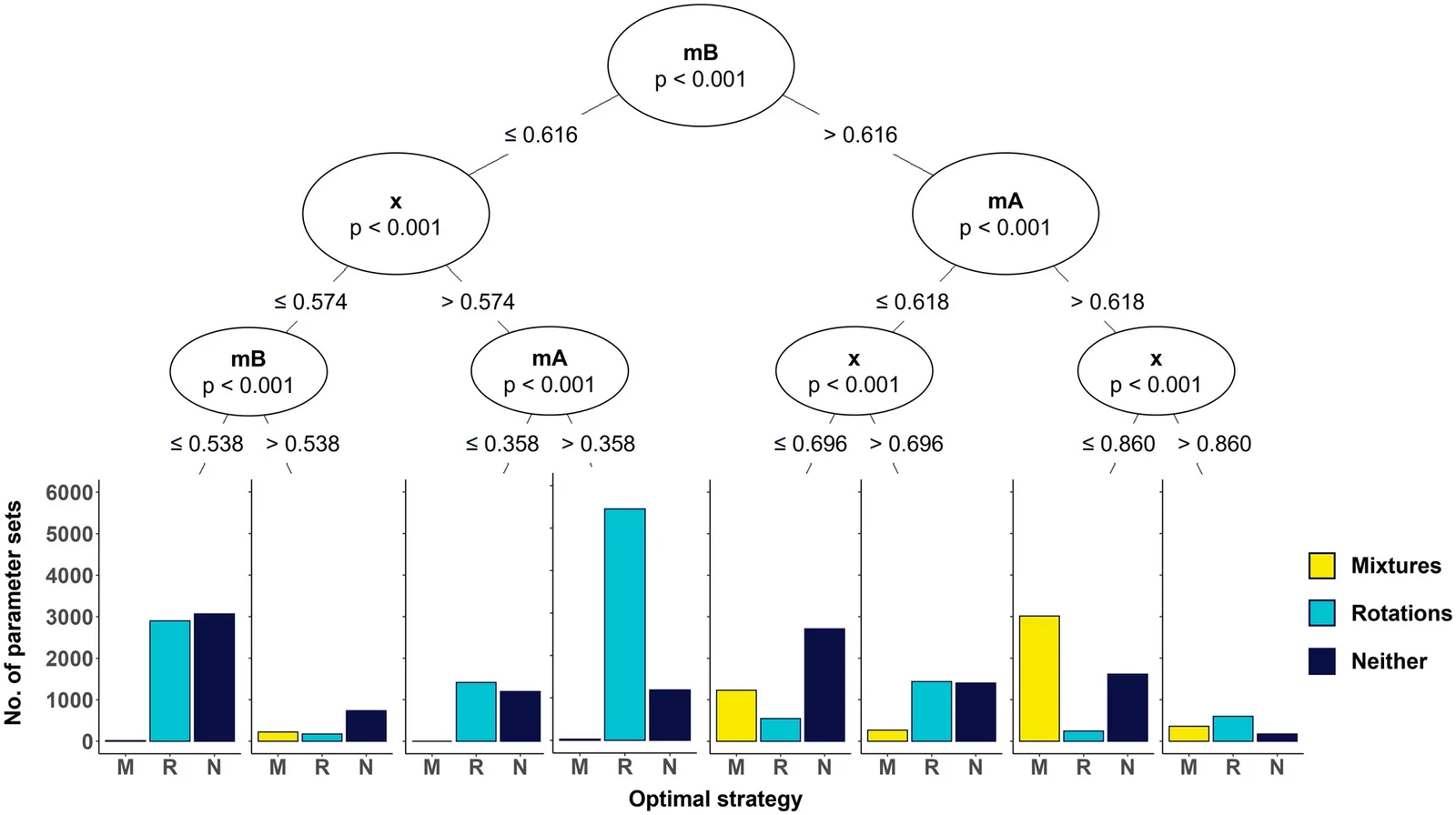
Decision tree analysis of parameter space preference for IRM strategies (mixtures or rotations) under the SAR model. mB—efficacy of pesticide B; x–exposure; mA—efficacy of pesticide A. Values displayed on the bar chart at each leaf node is the absolute number of parameter sets (from a total of 30,000) which fall into the parameter space segment as specified in the tree structure.

### Stochastic Genotypic Recombination

The SGR model extends the SAR model by allowing allele frequency distributions to deviate from HWE. The exact frequency distribution of the nine genotypes resulting from meiotic recombination during mating is preserved throughput the simulation. In general, the results of the SGR model resemble the SAR model, with similar optimal strategy distributions (Fig. 5A, rotations—58.40%, mixtures—38.43%) and similar categorical outcomes of resistance management (Fig. 5B). Time-to-fixation, probability-of-fixation, and probability-of-elimination favor rotations (Fig. 5C, D, and F), and time-to-elimination favors mixtures (Fig. 5E). Decision tree analysis shows almost identical results to SAR (Fig. 6 and Supplementary Fig. S3).

**Fig. 5.**
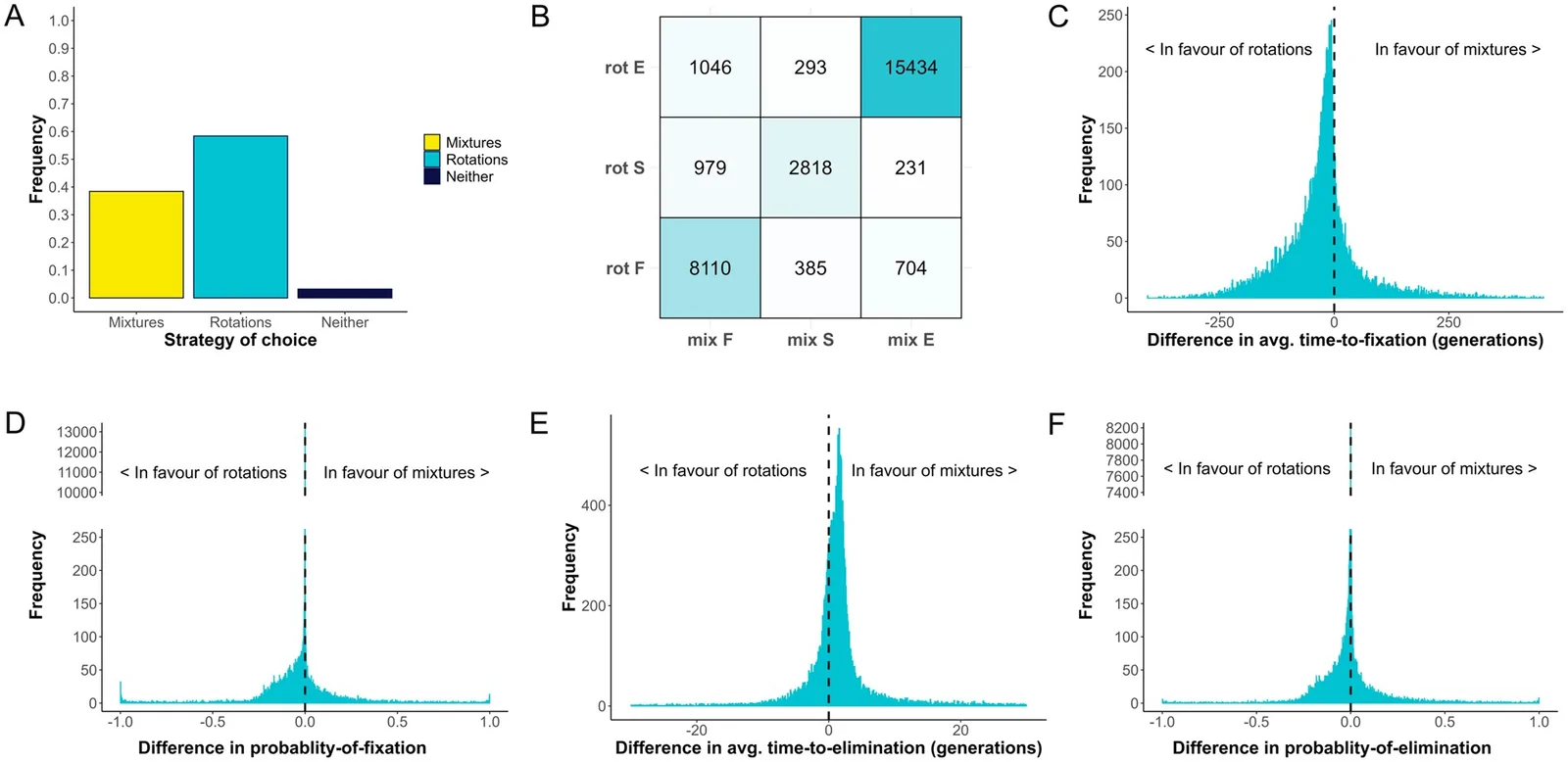
Overview of results from the stochastic genotypic assortment (SGR) model. (A) Overall preference for mixtures, rotations, or neither as an IRM strategy across the whole parameter space explored. The numerical value shown is the fraction of parameter sets which falls into each category under the SGR model. (B) The categorical resistance management outcome correlation matrix between mixtures and rotations under the SGR model. F—resistance allele reached fixation threshold of 0.5; S—resistance allele continued to spread at generation 500, it never reached frequencies of 0.5 or 0 within the simulation; E—resistance allele reached a frequency of 0 in the population and was eliminated. The numerical value shown is the absolute number of parameter sets which fell into each category out of all 30,000 parameter sets. (C) Conditional upon fixation, the difference between the number of generations taken for mixtures and rotations to fix the resistance allele under the SGR model. (D) The difference in probability-of-fixation between mixtures and rotations across all 30,000 parameter sets under the SGR model. (E) Conditional upon elimination, the difference between the number of generations taken for mixtures and rotations to eliminate the resistance allele under the SGR model. (F) The difference in probability-of-elimination between mixtures and rotations across all 30,000 parameter sets under the SGR model.

**Fig. 6.**
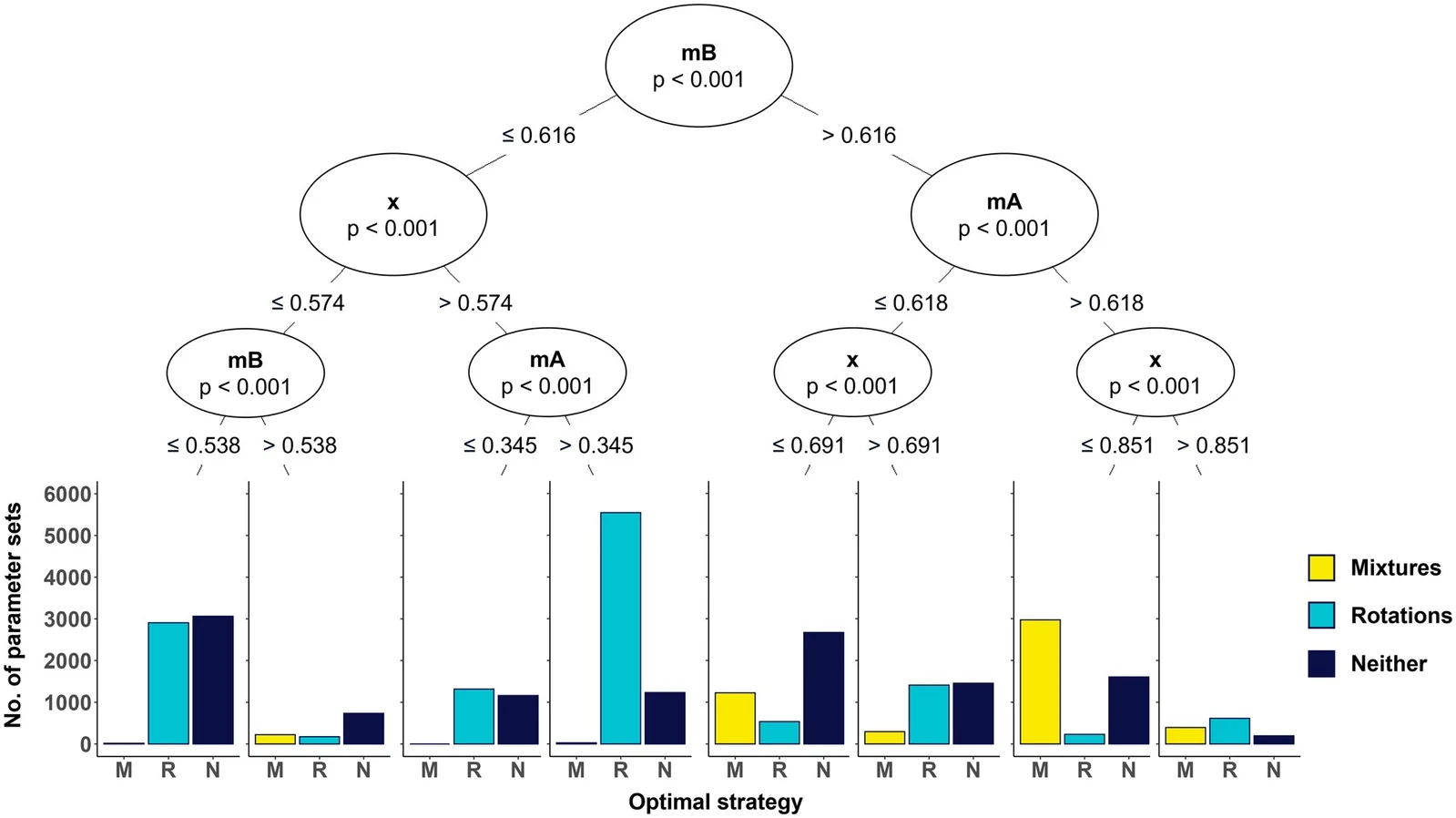
Decision tree analysis of parameter space preference for IRM strategies (mixtures or rotations) under the SGR model. mB—efficacy of pesticide B; x–exposure; mA—efficacy of pesticide A. Values displayed on the bar chart at each leaf node is the absolute number of parameter sets (from a total of 30,000) which fall into the parameter space segment as specified in the tree structure.

### The Effect of Stochasticity on Strategy Comparison

Stochasticity is introduced in the SAR model through multinomial sampling of a deterministically calculated zygote genotype distribution at every generation. This permits the loss of resistance alleles when they are rare.

Comparing the categorical outcomes between the DAR and SAR models, only 45.23% of cases show the same outcome when mixtures are applied (Fig. 7A), and 44.07% when rotations are applied (Fig. 7B). The inclusion of stochasticity can lead to resistance allele loss in cases where they are fixed under the DAR model. For instance, of the cases where mixtures lead to allele fixation in the DAR model, the modal outcome for 42.76% of these cases changes to allele elimination in the SAR model (Fig. 7A). A similar shift occurs in 42.21% of cases for rotations (Fig. 7B). A change from allele spreading to allele elimination occurs even more frequently, ranging from 71.91% for mixtures to 71.37% for rotations (Fig. 7A and B).

**Fig. 7.**
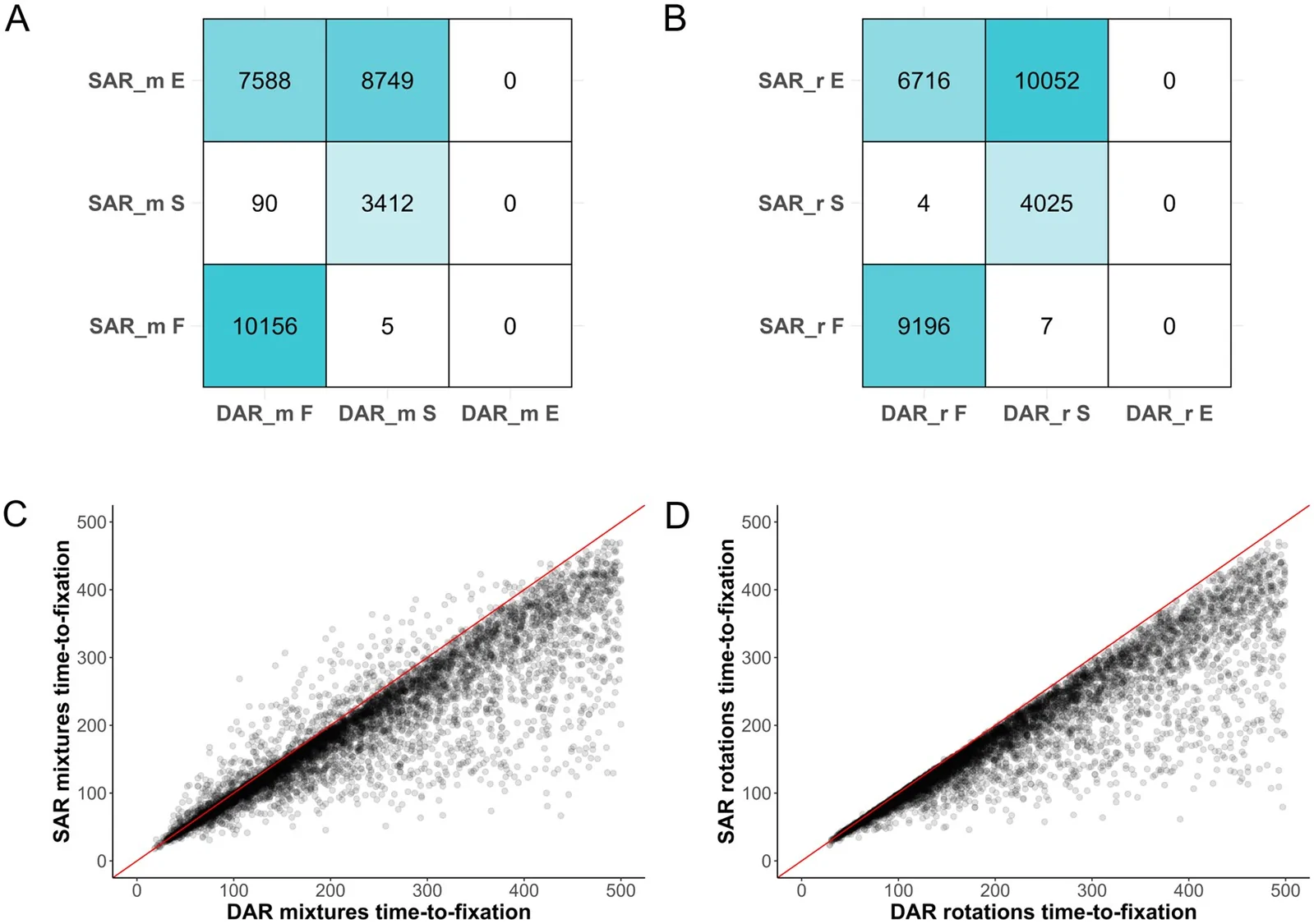
Comparison between the DAR and SAR model results to illustrate the effects of stochasticity on resistance management outcomes. (A) The categorical resistance management outcome correlation matrix between DAR and SAR when applying mixtures. F—resistance allele reached fixation threshold of 0.5; S—resistance allele continued to spread at generation 500, it never reached frequencies of 0.5 or 0 within the simulation; E—resistance allele reached a frequency of 0 in the population and was eliminated. The numerical value shown is the absolute number of parameter sets which fell into each category out of all 30,000 parameter sets. (B) The categorical resistance management outcome correlation matrix between DAR and SAR when applying rotations. The numerical value shown is the absolute number of parameter sets which fell into each category out of all 30,000 parameter sets. (C) Of the 7,588 parameter sets which switched from allele fixation in DAR to (most commonly) allele elimination in SAR, comparison of (mean) time-to-fixation when applying mixtures. (D) Of the 6,716 parameter sets which switched from allele fixation in DAR to (most commonly) allele elimination in SAR, comparison of (mean) time-to-fixation when applying rotations.

Decision tree analyses are carried out to understand the parameters driving this change. Using mixtures as an example, population size (k) emerges as the most important factor, with values below approximately 1.1 × 10^5^ being more likely to cause different resistance management outcomes between the SAR and DAR models (Supplementary Fig. S4A). As the population size increases, the DAR model becomes a better approximation of the SAR model since stochastic effects are diminished. When k is smaller than 1.1 × 10^5^, high values of x and $m_{A}$ also reduce the change in outcome, this can be explained as a highly selected-for allele is less prone to stochastic effects (Supplementary Fig. S4A). A similar pattern is observed for rotations, where stochasticity has the greatest effect when k is low, $f_{A}^{0}$ is low, x is high, and $m_{A}$ is high (Supplementary Fig. S4B).

For cases where the outcome shifts from fixation in DAR to elimination in SAR, the average time-to-fixation in SAR (conditional upon fixation occurring) is lower than in DAR (Fig. 7C and D). This is possible as the SAR time-to-fixation metric only includes a subset of stochastic repeats where stochasticity acted in favor of allele fixation, as opposed to the DAR metric, which is an average over all outcomes.

Overall, stochasticity significantly impacts the optimal strategy choice. As seen in Figs 1A and 3A, the inclusion of stochasticity favors mixtures. From Fig. 3E, it is clear that allele elimination occurs sooner with mixtures than with rotations. From Fig. 7A and B, stochastic simulations lead to many more allele elimination events than deterministic models. These two observations combined underlie the increased tendency to favor mixtures in stochastic simulations.

### The Effect of Assortment on Strategy Comparison

In the SAR model, genotype distributions are constrained by HWE, but this assumption is relaxed in the SGR model. The SGR model mimics meiotic recombination, allowing allele distributions to deviate from HWE proportions.

A high degree of agreement is observed between the SAR and SGR models, with over 99.7% of all parameter combinations showing the same modal outcome (Fig. 8A and B). This suggests that the effect of genotypic recombination on resistance evolution is much smaller than that of stochasticity. Further analysis shows that mixtures have a slight tendency to induce positive LD as measured by normalized Lewontin’s *D* (Supplementary Fig. S5A), whereas rotations do not generate LD in either direction (Supplementary Fig. S5B). However, the magnitude of such a difference is not sufficient for influencing the optimal strategy choice.

**Fig. 8.**
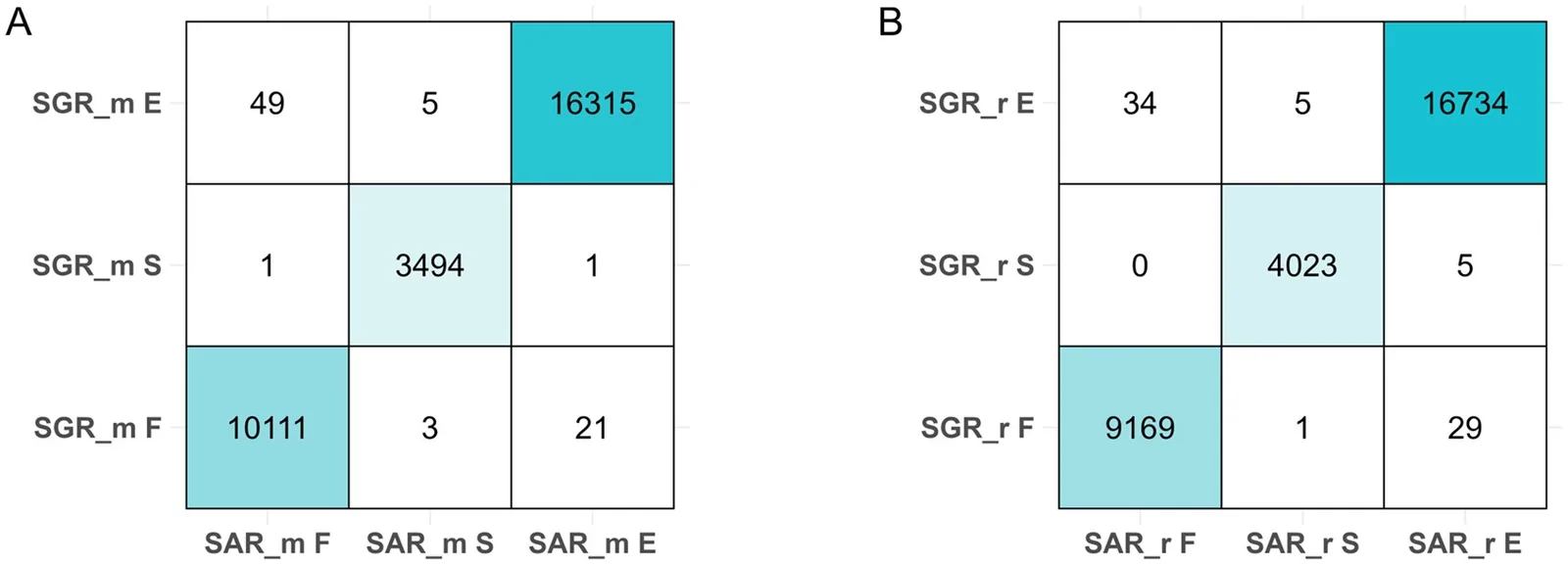
Comparison between the SAR and SGR model results to illustrate the effects of assortment on resistance management outcomes. (A) The categorical resistance management outcome correlation matrix between SAR and SGR when applying mixtures. F—resistance allele reached fixation threshold of 0.5; S—resistance allele continued to spread at generation 500, it never reached frequencies of 0.5 or 0 within the simulation; E—resistance allele reached a frequency of 0 in the population and was eliminated. The numerical value shown is the absolute number of parameter sets which fell into each category out of all 30,000 parameter sets. (B) The categorical resistance management outcome correlation matrix between SAR and SGR when applying rotations. The numerical value shown is the absolute number of parameter sets which fell into each category out of all 30,000 parameter sets.

Overall, genotypic assortment does not have a strong effect on resistance management strategies. The similar performance of mixtures in both models suggests that the SGR model does not significantly increase the formation of double-resistant individuals compared to what is assumed under HWE.

### Analytical Prediction of Simulation Outcomes

This section presents analytical predictions for key simulation outcomes in the SGR model, building upon the DAR and SAR models. It also includes solo-use simulations to isolate the effects of multi-pesticide applications.

### Analytical Predictions of Selection Coefficients

An initial prediction of solo-use dynamics under DAR assumptions shows that the analytical prediction for the selection coefficient can deviate from the simulation output when heterozygote-resistant individuals are more fit than homozygote-resistant individuals. Heterozygote advantage arises when the dominance of the resistance allele is higher than the dominance of its cost. Excluding these cases (∼17% of all parameter combinations) results in a good correlation between observed and predicted selection coefficients (Supplementary Fig. S6A).

This prediction is extended to rotations, where it is assumed that heterozygote resistance fitness is the primary driver of resistance spread. Excluding heterozygote advantage cases (∼17% of all parameter combinations), a good correlation is observed (Supplementary Fig. S6B). It should be noted that the application of heterozygote approximation analytics to multi-pesticide use implicitly assumes negative LD between the two resistance alleles under selection. Specifically, this means that resistance alleles spread through the population independently without interference, and any interactive effects would not be captured by such an approximation.

For mixtures, a similar assumption is made, and all parameter sets with heterozygote advantage relative to either resistant allele are excluded (∼47% of all parameter combinations). A good agreement is observed between simulated and predicted selection coefficients (Supplementary Fig. S6C). In a few cases, the analytical prediction underestimates the true selection coefficient, which is hypothesized to be a consequence of epistasis.

### Analytical Predictions in a Stochastic Solo-Use Model

Analytics are extended to predict key simulation outcomes such as probability-of-fixation and time-to-fixation in SAR solo-use simulations. Excluding heterozygote advantage cases, the probability-of-fixation is calculated using Kimura’s equation (Kimura 1957, Kimura 1962).

Supplementary Fig. S7A shows very good agreement between observed and predicted probability-of-fixation. A small number of cases show a positive predicted value when the observed value is zero, which is likely because the 500-generation limit in the simulation is too short for alleles with small effects to reach fixation, whereas the equation produces an “infinite-time prediction.” This highlights the importance of considering time-to-fixation alongside probability-of-fixation predictions.

For most solo-use cases, the predicted and simulation-derived time-to-fixation values align (Supplementary Fig. S7B). In a small number of cases, the allele reaches fixation faster than predicted. This is a consequence of stochastic dynamics: where allele fixation is not certain, the direction randomly chosen by stochasticity can result in either a speed up in resistance allele fixation or a speed up in resistance allele elimination. Since the simulation time-to-fixation is an averaged value conditional upon fixation, it appears that the time-to-fixation prediction (an average across all conditions) underestimates the true rate of resistance allele spread in these rare cases (Orr and Unckless 2014, Madgwick et al. 2024).

### Analytical Predictions for SAR and SGR Models

The analytical predictions for probability-of-fixation and time-to-fixation are extended to the full SAR model with mixtures and rotations. The predictions for mixtures align with expectations from the solo-use scenario (Supplementary Fig. S8A and B). For rotations, predictions for allele A also agree with expectations (Supplementary Fig. S8C and D). However, predictions for allele B underestimate the true probability of fixation in a significant number of cases (Supplementary Fig. S9A). Further analysis reveals these cases involve only two copies of resistance allele B at the start—the lowest value permitted in the simulation. Since one of the two pesticides is not applied in the first generation, there is an increased chance of eliminating these ultra-low-frequency alleles due to cost. To quantify this effect, a fitted linear model shows that the probability of allele elimination increases by 14.25% in these cases (Supplementary Fig. S9B). The good fit of the linear model indicates that this increase is constant across different parameter combinations. Adjusted probability-of-fixation estimates are then used to predict time-to-fixation (Supplementary Fig. S9C). These predictions are calculated assuming a rotation regimen which alternates compounds on a per-generation basis. Should this alternation interval increase, it may decrease the probability-of-fixation and therefore increase time-to-fixation. With a sufficiently long alternation interval, predictions for rotations would approach the solo-use scenario.

Finally, SGR model outcomes are predicted using the same analytics. The high similarity between SAR and SGR outcomes suggests that the underlying assumption—that the two resistance alleles undergo mostly independent selection—remains valid in the SGR model. The good agreement between SAR-based analytical predictions and SGR simulation outcomes (Fig. 9A–D) confirms that double mutants in the SGR model do not have a large effect on overall resistance evolution.

**Fig. 9.**
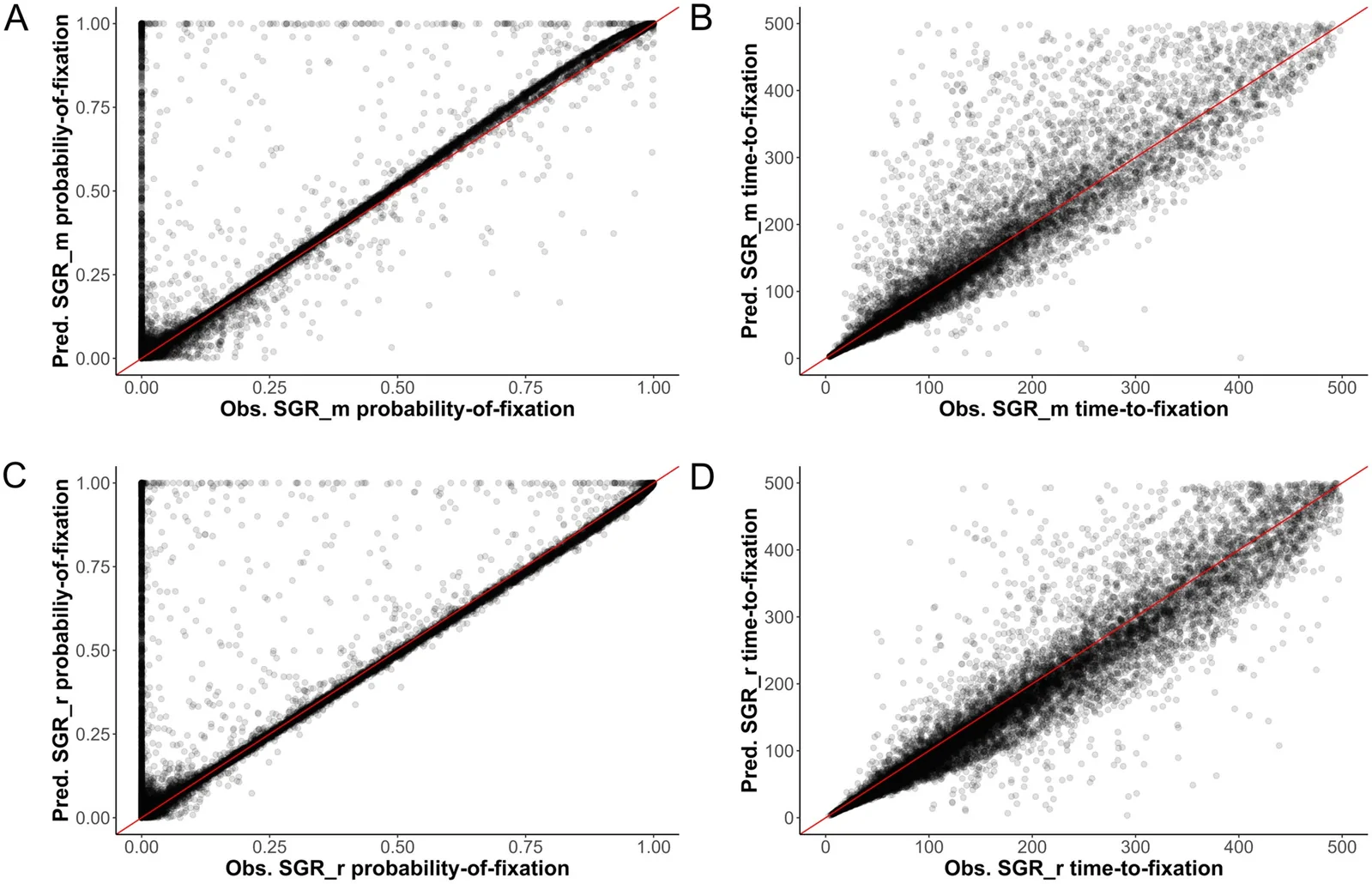
Analytical predictions of key simulation outcomes under the SGR model. (A) Comparison of simulation-observed vs. analytically predicted values of probability-of-fixation under the SGR model when applying mixtures. (B) Comparison of simulation-observed vs. analytically predicted values of time-to-fixation under the SGR model when applying mixtures. (C) Comparison of simulation-observed vs. analytically predicted values of probability-of-fixation under the SGR model when applying rotations. (D) Comparison of simulation-observed vs. analytically predicted values of time-to-fixation under the SGR model when applying rotations.

## Discussion

In the present study, three related population genetics models are constructed to comprehensively explore the possible effect of linkage disequilibrium (LD) on resistance management strategy choice in the general case where alleles are not biologically linked and compounds act independently. Specifically, mixtures and rotations are compared to address concerns that mixtures would be disfavored because they generate positive LD. The two components of how selection may build up LD, stochasticity and assortment, are examined separately. The DAR model follows conventional deterministic dynamics with alleles at HWE. The SAR model replicates most of the DAR model dynamics, with the exception that offspring generation incorporates multinomial stochasticity, but allele distributions are constrained to be at HWE at every generation. The SGR model replicates SAR model dynamics and additionally permits genotype distributions to deviate from HWE.

Decision tree analyses show that all three models find mixtures to be superior when exposure is low and pesticide efficacies are high, which is consistent with previous models (Mani 1985, Curtis and Otoo 1986, Roush and McKenzie 1987, Roush 1989, Levick et al. 2017). The introduction of stochasticity alone increases the favorability of applying mixtures over rotations. However, the addition of genotypic assortment does not have an appreciable effect on the choice of resistance management strategy. Indeed, the generation of accurate analytical predictions shows that the simulation results follow the expectations, assuming independent resistance allele selection and negative LD. Consequently, even when the SGR model permits LD to build up, the tendency for mixtures to generate a disproportionate number of double-resistant individuals is highly limited. Hence, in these simulations, the possibility of LD does not disfavor the use of mixtures for resistance management.

The rapid selection of the double-resistance genotype was first raised as a concern for mixtures by Curtis (1985), where it is argued that, should resistance alleles be dominant, any emergence of the double-resistant AaBb genotype would be rapidly selected for [see (Curtis 1985), Fig. 1], eliminating the benefit of applying mixtures. Filtering for parameter combinations where both resistance alleles are close to dominant ($h_{A}>0.85$, $h_{B}>0.85$) in our model, there is no strong evidence for the selection of double heterozygote individuals leading to rapid resistance spread. Allowing genotype distributions to deviate from HWE in the SGR model, a necessary condition for the rapid increase of AaBb genotype, no significantly different time-to-fixation is observed than in the SAR model, where such build-up of LD is not possible (Supplementary Fig. S10A).

Additionally, Comins’s prediction that LD will build up when the double heterozygote-resistant fitness (relative to the susceptible genotype fitness) is more than two times greater than the product of the two single heterozygote-resistant fitness values (Comins 1986) is also tested. Only 23 out of all 30,000 parameter combinations fulfill this criterion. With such a narrow parameter window, scenarios satisfying Comin’s criterion arise only under highly specific conditions; hence, they should be rare in a resistance management context. Among these 23 cases, no significant difference in mean time-to-fixation can be observed when mixtures are applied in the SAR or the SGR model (Supplementary Fig. S10B). Interestingly, 22 out of 23 cases have a mean time-to-fixation of less than 75 generations regardless of model choice. It is possible that Comins’s criterion favors parameter combinations with highly advantageous resistance alleles, which spread rapidly in the population. If this were the case, there would be insufficient time for LD to build up to a significant level before resistance frequencies reach the threshold of 0.5, at which point the pesticides employed would no longer be efficacious.

Concurrent to Curtis and Comins, Mani’s analysis of the impact of LD in resistance management focused instead on populations where resistance allele distributions do not begin at HWE (Mani 1985). He derived that any initial LD will be rapidly resolved with sufficiently high recombination (Mani 1985), a conclusion supported by current results. In the SGR model, when resistant alleles to each pesticide start to spread from single heterozygote individuals, there is negative linkage between the two resistance alleles at the beginning of the simulation. Whereas in the SAR and DAR models, starting with the same aggregate resistance allele frequencies, obeying HWE ensures that LD initially begins at 0. Aside from stochastic effects, the similarity of the SGR model results to the DAR model despite its initial negative linkage state supports Mani’s intuition of LD in resistance management.

Of the more recent discussions on resistance management, the results presented in this study are in broad agreement with recent theoretical models (Levick et al. 2017, Madgwick and Kanitz 2022b), with an additional stochastic dimension. Fundamentally, the lack of LD and associated effects can be explained by the choice of parameter space in the context of stochastic dynamics. In natural populations previously unexposed to pesticides, initial allele frequencies tend to be low in proportion to population size. Hence, it is unlikely that any double-resistant individuals exist at the beginning of the simulation, as would be expected for previously unexposed agricultural fields. From this starting point, resistance alleles must increase in frequency independently for some generations before double-resistant individuals are likely to emerge. If double resistance arises earlier through stochasticity, these rare genotypes are likely to be lost through random sampling. By the time double resistance becomes established in the population, both resistance alleles must already have reached appreciable frequencies independently. As resistance continues to spread exponentially, only a few generations remain before the 50% threshold is exceeded, leaving little opportunity for LD to accumulate and influence the eventual resistance management outcome.

This explanation agrees well with the accurate analytical prediction produced using heterozygote approximation in this study. The heterozygote approximation assumes that the single-resistant heterozygote fitness is the main determinant driving resistance fixation and probability (Comins 1977, 1986, May and Dobson 1986, Madgwick et al. 2024), and by extension assumes that resistance alleles spread independently even when multiple pesticides are employed. Here, it is shown that these assumptions remain valid when predicting mixtures resistance fixation and probability in an LD-permissive model, supporting the intuition that under the current model setup, resistance to both pesticides in a mixture predominantly spreads independently.

In sum, having taken inspiration from recently developed theoretical models of pesticide resistance management (Levick et al. 2017, Slater et al. 2017, Sudo et al. 2018, Helps et al. 2020, Madgwick and Kanitz 2022b), this study comprehensively explores the possible effects of LD in resistance evolution and optimal management strategy. Armed with three models which mapped out the effects of both stochasticity and genotypic assortment, the results show that assortment alone does not influence the behavior of mixtures application beyond that which imposed by stochasticity. Allowing LD to build up in the present context does not significantly alter the ability of mixtures to act as an effective resistance management strategy in delaying resistance spread, contrary to existing concerns (Curtis 1985, Sudo et al. 2018). It can thus be concluded that, under current assumptions, mixtures are a more robust strategy than previously believed.

## Supplementary Material

toag228_Supplementary_Data

## Data Availability

Code and data associated with this manuscript are available on Zenodo (Li 2026), at the URL: https://doi.org/10.5281/zenodo.21292916.
