# Supplementary material for: The effect of linkage disequilibrium on the evolution of resistance to multiple pesticides
Source: J Econ Entomol. 2026 Jul 27;119(5):3589–603. doi: 10.1093/jee/toag228 (PMC13658920; doi:10.1093/jee/toag228)
Supplement: Supplementary file 1 [file toag228_supplementary_data.zip › Supp. figure legends.docx]

**Supplementary figure legends**

Supp. Fig. 1. Decision tree analysis for DAR simulations with 0.9 fixation threshold and 1000-generation time horizon.

Supp. Fig. 2. Decision tree analysis for SAR simulations with 0.9 fixation threshold and 1000-generation time horizon.

Supp. Fig. 3. Decision tree analysis for SGR simulations with 0.9 fixation threshold and 1000-generation time horizon.

Supp. Fig. 4. Decision tree analysis for understanding parameter contributions to the concordance or discordance of resistance management outcomes between DAR and SAR. A) Mixtures. B) Rotations.

Supp. Fig. 5. Comparison of normalised Lewontin’s D between mixtures and rotations in SGR, non-zero median of 10,000 stochastic repeats shown. A) Mixtures, resistance allele A. B) Rotations, resistance allele A.

Supp. Fig. 6. Analytical predictions of selection coefficients, simulation-observed vs. predicted. A) DAR solo-use. B) DAR rotation. C) DAR mixtures.

Supp. Fig. 7. Analytical predictions of simulation metrics for solo-use scenarios, simulation-observed vs. predicted. A) DAR solo-use, time-to-fixation. B) SAR solo-use, probability-of-fixation. C) SAR solo-use, time-to-fixation.

Supp Fig. 8. Comparison of simulation-observed metrics under the SAR model against analytical predictions. A) Mixtures, probability-of-fixation. B) Mixtures, time-to-fixation. C) Rotations, probability-of-fixation. D) Rotations, time-to-fixation.

Supp Fig. 9. Analytical predictions of simulation metrics for allele B under the SAR model when applying rotations. A) Probability-of-fixation, without correction. B) Probability-of-fixation, corrected for increased chance of resistance allele loss if resistance allele started at one homozygote copy in the population. C) Time-to-fixation, calculated using the corrected probability-of-fixation.

Supp Fig. 10. A) Comparison of mixtures time-to-fixation across the SAR and SGR models when both resistance alleles are close to full dominance (hA > 0.85 & hB > 0.85). B) Comparison of mixtures time-to-fixation across the SAR and SGR models when the fitness of the double heterozygote ($wAaBb$) is more than twice as large as the product of the fitness of the single heterozygotes ($wAabb\times waaBb$). Recombination ($r$) is assumed to be 0.5.
